# Unmasking Risky Habits: Identifying and Predicting Problem Gamblers Through Machine Learning Techniques

**DOI:** 10.1007/s10899-024-10297-4

**Published:** 2024-04-03

**Authors:** Máté Cs. Sándor, Barna Bakó

**Affiliations:** Institute of Economics, Corvinus University of Budapest, Fővám tér 8, 1093 Budapest, Hungary

**Keywords:** Machine learning, Problem gambling, Identification, Prediction

## Abstract

The use of machine learning techniques to identify problem gamblers has been widely established. However, existing methods often rely on self-reported labeling, such as temporary self-exclusion or account closure. In this study, we propose a novel approach that combines two documented methods. First we create labels for problem gamblers in an unsupervised manner. Subsequently, we develop prediction models to identify these users in real-time. The methods presented in this study offer useful insights that can be leveraged to implement interventions aimed at guiding or discouraging players from engaging in disordered gambling behaviors. This has potential implications for promoting responsible gambling and fostering healthier player habits.

## Introduction

The gambling industry’s rapid technological transformation has led to unprecedented accessibility, contributing to a concerning rise in problem gambling cases (Potenza et al., 2011; Chagas & Gomes, 2017). Although the recent pandemic initially reduced overall gambling participation, it triggered a surge in online and problem gambling, with younger individuals disproportionately affected (Wardle et al., 2021; Hodgins & Stevens, 2021). The societal costs associated with problem gambling are projected to have a profound impact on the economy (Hofmarcher et al., 2020). Notably, online gambling platforms employ persuasive tactics called "sludges" to entice users to engage in longer and riskier betting practices (Newall, 2019; Newall et al., 2020). Moreover, the industry utilizes industrial machine learning solutions to support these practices (Coussement & De Bock, 2013), and the utilization of dark patterns has demonstrated significant effects on consumer manipulation (Bogliacino et al., 2023). Consequently, regulatory bodies have initiated investigations into the adverse implications of online choice architecture.

To address the issue of problem gambling, various studies have examined the effectiveness of nudges, such as implementing loss limits and providing personalized feedback, in discouraging addictive behaviors (Brodeur, 2019; Auer et al., 2018; Auer & Griffiths, 2020). Promising results have been observed in brick-and-mortar casino gambling through the introduction or promotion of self- and forced exclusion periods (Kotter et al., 2018). In the case of online gambling, interventions that disrupt the gambling flow, such as fixed or self-defined monetary limits, have shown effectiveness (Folkvord et al., 2019). However, results by Caillon et al. (2019); Giroux et al. (2017) suggest that the effectiveness of these measures in online gambling remains unclear.

Unsupervised machine learning techniques have been successfully used to identify vulnerable user groups in gambling (Deng et al., 2019; Braverman & Shaffer, 2012; Xuan & Shaffer, 2009). Machine learning algorithms can also predict the development of addictive patterns (Mak et al., 2019). Previous studies on gambling data have effectively predicted self-exclusion using supervised learning techniques like logit regression, gradient boosting, and neural networks (Percy et al., 2016; Ukhov et al., 2021; Buttigieg et al., 2022; Finkenwirth et al., 2021), relying on observed behavioral markers like frequency of play, risk-taking behavior, and bet sizes. However, one limitation of previous studies is their reliance on rule-of-thumb measures to select the target subgroup of gamblers. This approach may introduce researcher bias and hinder the transferability and general efficacy of the results across different game types and designs.

In this analysis, we propose a new approach that avoids using pre-observed labeling information, thereby reducing potential bias towards self-aware gamblers. Instead, we combine unsupervised machine learning techniques to create labels for problem gamblers. Once the target categories are established, we simplify the process of selecting specific prediction algorithms using automatic machine learning (autoML) algorithms. This approach ensures a more objective and robust method for identifying problem gamblers and predicting their behavior.

## Methods

In this study, our main goal is to demonstrate the effectiveness and ease of predicting problem gambling. To achieve this, we adopt a dual approach. First, we employ k-means clustering to categorize our target users based on their gambling behavior over a 7-day period, following an initial 3-day period. This clustering process helps us assign labels to our problem gambling group. Next, we develop predictive models that can forecast the cluster label of each player based on their behavior during the initial 3-day period. To accomplish this, we utilize a large dataset of betting transactions extracted from publicly available data sources.^1^

### Dataset

Among Bitcoin, the pioneering decentralized digital currency, as one of the early use cases, online gambling emerged as a prominent application. Bitcoin’s innovative system provided an ideal environment for experimentation, and due to its unregulated nature, numerous online gambling sites have sprung up since 2012, leveraging the Bitcoin ecosystem. One of the most successful ventures within the cryptocurrency community was Satoshidice.^2^ This platform implemented a simple yet fair gambling system, offering games to players with varying odds or levels of risk. The fairness of the games was ensured through two mechanisms: the expected return for each game was fixed, thereby creating a house cut that remained independent of the risk level. Additionally, the game outcomes were determined by a "dice roll" generated by combining information from the Bitcoin ledger related to the bet itself and a pre-set secret, which could be independently verified by the players.

The game process was straightforward. Players selected their desired level of risk by choosing a specific game from a predefined list, which presented various winning probabilities (inversely proportional to the odds) alongside a unique wallet address. By initiating a transaction to one of these addresses, the player placed a bet with the sent amount (within specific bet limits). The site assessed the bet based on transaction details and the secret key, promptly sending a return transaction reflecting the outcome. Although blockchain confirmation times in 2013 typically ranged from 5-7 minutes, most bets received instantaneous responses from the site. Given the blockchain’s public nature, it is possible to extract a comprehensive history of all incoming and outgoing transactions associated with any address on the network. We collected all bets placed at and return transactions sent by Saoshidice during its operational period in the specified form (the site transitioned to a prepay system in 2014). Our dataset comprises a complete longitudinal observation set of betting transactions, with five 21-day periods used to assess the robustness of our procedure over different samples and timeframes. For detailed information on the data gathering methodology and resources, see Bako and Sándor (2021) (Table 1).^3^.

**Table 1:** Summary statistics of the subsets of the observed gambling history used

	Start	Number	Number	Total bets	Median bet	Mean daily
	date	of bets	of users	placed	size	price
				(BTC)	(BTC)	(USD/BTC)
A	2012-05-02	119,399	1002	46,649	0.04	5
B	2012-09-17	129,265	2114	85,280	0.06	12
C	2012-12-17	252,301	3405	407,140	0.04	13
D	2013-05-04	329,155	3432	100,430	0.02	111
E	2013-09-11	86,520	1400	62,920	0.03	123

The exchange rates have been sourced from the public historical data published by the online cryptocurrency exchange aggregator BitCoinCharts (see http://www.bitcoincharts.com)

From the transaction details, we can directly observe the following descriptors:

- **Player ID**: User identification label created based on the dataset of Kondor et al. (2014). The ID links transactions associated with the Bitcoin addresses controlled by the same entity. However, it does not provide any personal or location information about the player in question.
- **Time of bet**: Timestamp given to the Bitcoin transaction of the bet placed.
- **Time of answer**: The timestamp is assigned to the answering Bitcoin transaction, which we have paired with the bet.
- **Game ID**: The game selected by the player is determined by the target of the betting transaction. Directly linked to this target, we can assign a fixed winning probability and odds to the respective bet. This enables us to determine the specific game being played and the associated chances of winning for each betting transaction.
- **Bet amount**: The part of the bet transaction that has been directed towards the selected game address.
- **Answer amount**: The amount of Bitcoin directed back to the betting addresses from the SatoshiDice wallet determines the outcome of the gamble. This return transaction reflects the winnings or losses of the bet and makes it possible to determine the final result of the gambling activity.

From the variables mentioned above, we can derive several informative descriptive measures of the gambling process. While one approach could involve treating this data as a time series, as demonstrated in Peres et al. (2021), we find that producing daily aggregates achieves similar clustering outcomes without the computational complexity associated with the former method.

To facilitate both the labeling and predicting exercises, we have derived the following aggregates. It’s worth noting that these aggregates largely align with the observed behavioral markers used in previous studies (Deng et al., 2019). By employing these derived measures, we can effectively capture important aspects of the gambling behavior and use them to categorize and predict the behavior of interest.

- **Number of games**: Number of bets placed on the given period, transformed to a logarithmic scale.
- **Number of days active**: Number of calendar days that the player placed bets from the observed period (only used for labeling).
- **Number of sessions per days active**: Number of game sessions played defined by successive bet chains where no more than 1 hour has been spent inactive by the user, divided by the number of active days.
- **Median winning probability**: Median of the implied winning probability of the bets placed during the period. This describes the risk appetite of the player.
- **Range of winning probability**: Distance of the smallest and largest implied winning probability of the bets placed during the period. This describes the variability in risk taken by the player.
- **Mean bet**: Mean of the bet amounts placed during the period (in BTC). A logarithmic transformation has been applied.
- **Maximum bet**: Maximum of the bet amounts placed during the period (in BTC). A logarithmic transformation has been applied.
- **Total payout**: The aggregated amount of bets placed and answers received by players during the period (in BTC) resulting in the total gains/losses.

Our analysis consists of two main steps, with the second step involving the prediction of labels created in the first step. To facilitate this process, we establish two distinct subsets from each of our samples. What sets our approach apart is that we use shorter sample durations for both clustering and prediction compared to previous studies such as Braverman and Shaffer (2012) or Xuan and Shaffer (2009), which typically relied on 30-day to full history samples. For each gambler in each sample, we identified a 10-day period starting from their first betting day in the given sample. This window was then divided into the first 3 days and the last 7 days. The last 7-day window was utilized to identify emergent behavioral patterns indicative of problem gambling tendencies. On the other hand, the first 3-day window served as the basis for predicting the labeling of problem gambling behavior.

To create the clustering dataset, we aggregated relevant variables over the week-long window. Conversely, for the prediction dataset, we aggregated the data on a daily basis. Additionally, we introduced additional variables representing the change over days in the number of daily bets and the mean bet size. These features are crucial for predicting problem gambling labels effectively. By employing shorter sample duration and employing different aggregation methods for clustering and prediction, we demonstrate the robustness and efficiency of our approach. This allows us to effectively identify and predict problem gambling behaviors with improved accuracy and computational efficiency compared to previous studies.

### Labeling Problem Gamblers: Unsupervised Learning

K-means clustering is a widely used method in behavioral profiling, employed in marketing (Arumawadu et al., 2015), psychological settings (Stegmann et al., 2019), and specifically in analyzing gambling behavior (Braverman & Shaffer, 2012; Xuan & Shaffer, 2009). The key advantage of this unsupervised method is that it provides an unbiased separation of players based solely on their gambling profiles, devoid of any influence from researchers or regulators.

In our analysis, we use one week-long aggregates of the measures presented in Section 2.1. This observation period begins three days after the players’ first observed betting day. It’s worth noting that inclusion in this set indicates that players placed bets between the third and tenth day after their first bet in the sample. The user retention rate, as observed in this manner, varies between $14\%$ (sample C) and $35\%$ (sample E). Spearman correlations between the input variables generally stay below $r<.6$. Slightly higher correlations ($.6< r < .8$) are observed between the mean logarithmic bet versus the maximum bet and the number of active days versus the number of games played. However, deviations from this linear trend are significant in both separation and later prediction, indicating substantial variations in bet amounts and activity levels. We acknowledge the presence of outliers in our dataset (e.g., extreme number of bets or extremely large maximum bets), which can impact the robustness of k-means clusters. To address this, we employ the method of trimmed k-means clustering (Cuesta-Albertos et al., 1997; Hennig, 2020), allowing for a $1\%$ trimming factor, ensuring high stability for the specific separation we are focusing on. Based on measurements of both the Silhouette and Dunn indices, the optimal cluster number for all samples is found to be two.

### Predicting Gambling Behavior: autoML

Our prediction process involves categorization, where various techniques can be used, such as generalized linear models, random forests, gradient boosting, and deep learning algorithms. However, manually detailing and fine-tuning these methods to find the optimal one (or ensemble) for the given problem can be cumbersome. Instead, we demonstrate a more efficient approach using automatic machine learning techniques, specifically H2O’s autoML package (LeDell & Poirier, 2020; LeDell et al., 2022). This approach allows us to find the optimal model (or combination) by leveraging goodness-of-fit measures. By using autoML, we streamline the model selection process, producing robust and cross-validated models. This automation not only saves time but also ensures a reproducible process that can be easily deployed into production and archived for future reference or investigation.

In our prediction process, we have two targets: user retention, which predicts whether the observed player will continue placing bets in the target period or leave the game, and identification of players belonging to the group labeled as "intensive" during the clustering phase, indicating signs of problem gambling. To train the model, we use the variables described in Section 2.1, aggregated on a daily basis for the 3-day gambling period of our users starting from their first betting day in our samples. We run the autoML algorithm with default settings, including 5-fold internal cross-validation, creating 10 model sets, and a computational limit of 300 seconds. The calibrations are performed on a desktop computer without GPU support. This automated approach ensures an optimized model selection process and facilitates efficient and accurate predictions for both user retention and problem gambling identification.

## Results

### Labeling

Table 2 presents the median values of the input variables for the identified clusters. A clear contrast is evident for most of these measures between the casual (-) and intensive (+) groups. The most notable difference lies in the dimensions of gambling frequency: the intensive group places significantly more bets (ranging from 62 to 303) compared to the casual group (ranging from 6 to 7). Furthermore, members of the intensive group engage in gambling almost every day during the observation period, while casual players only participate for 1 to 2 days. Additionally, the intensive group returns to betting multiple times a day, with the number of daily sessions exceeding 2.

Analyzing risk-taking behavior, we observe that both groups often opt for "balanced" bets, offering approximately 50% probability of winning (or a multiplier of 2). However, the intensive group displays a much wider variation in risk-taking compared to the casual group. A similar pattern is noticeable for bet sizes. While the average bet sizes might not differ significantly, the maximum bets placed by players in the intensive group tend to be approximately an order of magnitude higher on average. The difference in expected losses (total payout) is a direct consequence of the aforementioned observations. Since the game is implemented fairly, with the house cut independent of the wager’s risk level, players in the intensive group, who engage in more frequent and higher-risk betting, can expect to accumulate larger losses on average.

In summary, the identified clusters exhibit distinct behavioral patterns, with the intensive group demonstrating higher gambling frequency, risk-taking, and bet sizes, resulting in higher expected losses due to the nature of the game’s fairness.

**Table 2:** Median (IQR) statistics of the clusters identified in the data samples (described in Table 1)

	Group	Games	Days	Sessions	Median risk	Risk range	Mean bet	Max bet	Total payout
A	-	6 (4)	2 (2)	1.5 (0.8)	48.8 (25.0)	0.0 (36.6)	0.07 (13.53)	0.18 (10.00)	-0.10 (0.44)
	+	89 (5)*	6 (3)*	2.8 (1.0)*	48.8 (24.4)	42.5 (52.6)*	0.13 (18.52)^†^	2.00 (7.98)*	-0.33 (3.67)
B	-	7 (6)	1 (2)	1.5 (1.0)	48.8 (28.1)	0.0 (24.4)	0.05 (12.81)	0.20 (10.00)	-0.02 (0.42)
	+	99 (6)*	6 (3)*	2.1 (1.4)*	48.8 (36.6)	48.8 (48.8)*	0.15 (10.08)^†^	2.48 (9.87)*	-0.57 (6.78)
C	-	9 (7)	2 (2)	1.5 (1.0)	50.0 (24.4)	1.2 (32.6)	0.07 (17.79)	0.37 (40.00)	-0.04 (0.68)
	+	303 (8)*	6 (3)*	2.3 (1.2)*	48.8 (13.4)	71.7 (47.3)*	0.05 (9.12)	2.56 (9.43)*	-0.70 (7.79)^†^
D	-	7 (7)	2 (3)	1.5 (1.3)	48.8 (13.4)	6.1 (67.1)	0.02 (5.80)	0.04 (9.29)	-0.03 (2.45)
	+	105 (5)*	6 (2)*	2.6 (1.0)*	48.8 (37.8)^†^	48.7 (36.6)*	0.04 (3.33)^†^	0.85 (9.05)*	-0.32 (0.12)
E	-	6 (5)	2 (2)	1.6 (0.8)	48.8 (54.9)	0.0 (25.6)	0.02 (7.33)	0.04 (12.31)	-0.02 (0.18)
	+	62 (5)*	6 (3)*	2.5 (1.4)*	48.8 (31.7)	50.0 (40.0)*	0.03 (5.28)^†^	0.47 (14.27)*	-0.15 (1.81)

The groups labeled to be the intensive gamblers are signed with a + in the group column and highlighted with grey background. The results of Kruskal-Wallis rank sum tests for difference between the group descriptors are signed on the intensive group values (P-levels: $\dagger .05, *.001$). The separation of the groups is consistent in the dimensions of game frequency (games, days active and sessions per day) and also the wider risk and bet size range

**Table 3:** Descriptors of prediction performance of top models found using the autoML method

Retention prediction							
	Sample size	Prevalence	Best model	Models in ensemble	AUPCR	Logloss	RMSE
A	445	34%	GBM		0.92	0.20	0.25
B	1200	18%	Ensemble	DL/DRF/GBM/GLM	0.88	0.11	0.18
C	1837	14%	Ensemble	DRF/GBM/GLM	0.91	0.09	0.16
D	2263	30%	Ensemble	DL/DRF/GBM/GLM	0.91	0.16	0.22
E	741	35%	GBM		0.90	0.18	0.23
Intensive period prediction							
	Sample size	Prevalence	Best model	Models in ensemble	AUPCR	Logloss	RMSE
A	445	17%	GBM		0.70	0.23	0.27
B	1200	10%	Ensemble	DL/DRF/GBM/GLM	0.63	0.15	0.22
C	1837	6%	GBM		0.58	0.11	0.19
D	2263	14%	GBM		0.74	0.19	0.25
E	741	16%	GBM		0.62	0.25	0.29

Sample size shows the number of players who have placed a bet in the first 12 days of the sample. Prevalence refers to the relative size of the target group compared to the sample size. The models used are gradient boosting (GBM), deep learning (DL), distributed random forest (DRF) and generalized linear (logit) model (GLM). Submodels are only detailed for ensembles

### Prediction

The top section of Table 3 displays the predictive performance of the best models generated by the autoML algorithm for all our samples. The results reveal remarkably high area under the curve (AUC) measures and low errors, alongside satisfactory log loss compared to the target prevalence. These findings indicate that, on average, we can accurately predict whether a player will or will not place a bet in the 4th to 10th day following their initial betting day, based on the optimal probability level set. This high accuracy in predictability of user retention is not surprising since modeling this metric has already become an industry standard, hence yielding expectedly strong results.

Looking at the lower part of Table 3, we observe the same statistics for predicting player inclusion in the intensive clusters, as described in Section 3.1. Comparing this prediction to the user retention case, we notice a slightly weaker predictive strength, but the metrics still demonstrate good predictive quality. The area under the curve metrics remain very high, and the log losses are significantly below trivial levels. With the optimal probability threshold, these models provide categorization with only a few instances of mislabeling for each sample. These models exhibit explanatory power similar to recent analyses, as seen in Finkenwirth et al. (2021). In most cases, gradient boosting models performed the best as standalone models, and ensembles of gradient boosting and other models were used in other instances. It’s worth noting that during the autoML training, a set of alternative methods (both standalone and ensemble) were provided, and they exhibited comparable performance levels. The high predictive quality of these models, even in standalone configurations, highlights their robustness and effectiveness in identifying players likely to belong to the intensive gambling clusters.

## Conclusion

The successful demonstration of the effectiveness of unsupervised learning methods in separating players exhibiting signs of problem gambling has significant implications for the field of responsible gambling and player protection. By identifying key variables that measure the intensity of gambling, such as the number of bets placed and the frequency of betting sessions, we can easily detect the group displaying problem gambling attitudes. This separation process has proven to be robust and reliable across various observation periods, even when dealing with varying sample sizes, making it a valuable and adaptable tool for early identification of problem gambling behaviors.

The ability to apply the chosen behavioral descriptors to different types of gambling, regardless of their specific structures, highlights the potential universality of this approach. This flexibility allows for the assessment of problem gambling tendencies in various gambling contexts, providing valuable insights for policymakers, regulators, and gambling operators. However, there are certain manual steps involved in the process, which may vary when dealing with other types of gambles. Determining the optimal number of groups for separation and subsequent labeling requires careful consideration and domain-specific knowledge. Additionally, the lack of a follow-up measure to validate whether the identified players are indeed problem gamblers may lead to lower labeling accuracy for true problem gamblers. Future research should focus on incorporating follow-up measures to enhance the accuracy and reliability of player categorization.

Machine learning approaches, such as the ones used in this study, offer an easy-to-implement monitoring tool for gambling platforms. These models can serve as a foundation for implementing proactive measures, such as nudging or forced exemptions, to deter at-risk gamblers from developing or continuing problem gambling behaviors. By identifying players early on who show signs of problematic gambling, operators can provide targeted interventions and support to promote responsible gambling practices and minimize harm. It is essential to recognize that the effectiveness of forced exemptions hinges on their widespread application on a market-wide scale. This measure prevents problem gamblers from simply shifting to other gambling venues or online sites, ensuring a more comprehensive and effective approach to player protection.

While the results of this study are promising, further replication and validation on other forms of gambling, such as online versions of classical casino games and sports betting, are necessary to assess the generalizability of the findings.^4^ Conducting a control group study with real gamblers, along with follow-ups and psychological profiling, would provide valuable data to compare the effectiveness of player selection and the optimal combination of nudging or forced deterring techniques. This comprehensive investigation would yield deeper insights into the potential impact of these interventions on curbing problem gambling and fostering responsible gambling practices on a broader scale.

## Footnotes

1 Scripts used for data preparation and analysis are made publicly available at github.com/sampaat/problem_gambler_prediction.

2 see https://web.archive.org/web/20121103121459/http://www.satoshidice.com/

3 The dataset used for the analysis can be accessed at DOI: 10.5281/zenodo.5600259.

4 Another limitation of our analysis is that the identification of risky behavior relies on specific past behavior which may not be available or observable. In such cases, a method presented in Codagnone et al. (2020) could provide promising results.
